# A systematic review of the effects of diet on the gut microbiota in individuals at elevated risk for colorectal cancer

**DOI:** 10.3389/fnut.2026.1818522

**Published:** 2026-10-16

**Authors:** Saurabh Pandey, Jenna Skidmore, Jaapna Dhillon

**Affiliations:** 1 Division of Food, Nutrition, and Exercise Sciences, College of Agriculture, Food and Natural Resources, University of Missouri, Columbia, MO, United States; 2 Department of Nutrition and Exercise Physiology, School of Medicine, University of Missouri, Columbia, MO, United States

**Keywords:** colon cancer, colorectal cancer, CRC, dietary intervention, gut microbiome, gut microbiota, rectal cancer, metabolomics

## Abstract

**Background:**

Colorectal cancer (CRC) poses a major public health concern, with emerging evidence highlighting the critical role of the gut microbiota in CRC progression. Considering that diet is a major modifiable factor influencing CRC risk, partly through its interaction with the gut microbiome, this systematic review evaluated the effects of dietary intervention RCTs, including supplements, functional foods, whole foods, and dietary patterns, on the gut microbiome in elevated-risk individuals.

**Methods:**

A search of PubMed, Scopus, and Web of Science, and manual sourcing of systematic reviews, meta-analyses, and reference lists of relevant papers identified 5,558 records.

**Results:**

Following screening, seven studies met inclusion criteria, focusing on microbial diversity and taxonomic composition. Findings demonstrate that effects on microbial diversity metrics were minimal and were observed only with a general healthy eating dietary pattern, navy bean powder interventions, and with one rice bran intervention. No consistent patterns emerged by intervention type (functional compounds, whole foods, or dietary pattern), intervention duration, or by diversity metric. In contrast, genus-level compositional shifts were more frequently reported, although directionality varied by dietary exposure. Enrichment of Faecalibacterium, Bifidobacterium, and Lactobacillus was reported in some rice bran and navy bean interventions, whereas reductions in Akkermansia, Ruminococcus, Bacteroides, Prevotella, and Porphyromonas were observed in others. Among the four trials assessing microbial-derived metabolites, some were associated with increased short-chain fatty acids and plant-derived metabolites while others reported reductions in secondary bile acids, p-cresol sulfate, and branched-chain bacterial fatty acids.

**Conclusion:**

Overall, this review highlights the potential of dietary strategies to modulate the gut microbiome in populations at risk for CRC, while also addressing critical gaps in the current evidence and offering direction for the development of evidence-based dietary interventions that incorporate microbiome assessment. Limited RCTs and heterogeneity in outcomes limit generalizability. Future research should conduct rigorous RCTs across the lifespan, using microbiome and metabolite analyses such as shotgun metagenomics, meta-transcriptomics, or metabolomics and examining understudied dietary patterns to guide CRC prevention.

**Systematic review registration:**

https://www.crd.york.ac.uk/PROSPERO/view/CRD42024530038, identifier CRD42024530038.

## Introduction

Colorectal cancer (CRC) is one of the most frequently diagnosed cancers globally and accounts for a considerable proportion of cancer-associated illness and death, creating a substantial public health burden (1). Recent projections indicate a dramatic rise in CRC incidence among young adults, with expected increases of up to 124% for those aged 20–34 and nearly 46% for individuals aged 35–49 by the year 2030 (2). Globally, based on GLOBOCAN 2022 estimates, CRC incidence is three to four times higher in developed countries than in developing regions, reflecting its association with socioeconomic development (3).

Diet plays a pivotal role in CRC risk influencing its development through interactions with the gut microbiome (4). Adherence to dietary patterns such as the Mediterranean diet, Healthy Eating Index-2005, or prudent dietary patterns, and other patterns rich in fruits, vegetables, and whole grains, is associated with reduced CRC risk (relative risk [RR] 0.45–0.90) (5). Conversely, dietary patterns dominated by processed and red meats, refined foods, and Western diets are linked to significantly increased CRC risk (RR 1.18–11.7). Similarly, cohort and case–control studies in young adults at risk for early-onset CRC show that diets high in sugar-sweetened beverages, deep-fried foods, and low in folate and fiber are associated with increased cancer risk, while fruit and vegetable-based diets rich in micronutrients are protective (6).

The gut microbiome mediates diet-related effects on CRC risk. Dysbiosis, or a disruption in microbial balance, contributes to chronic inflammation, genotoxic metabolite production, and immune dysregulation, all of which are implicated in CRC development (4, 7, 8). For instance, high fat diets have been shown to stimulate specific gut microbiota to produce carcinogenic secondary bile acids, which can promote CRC development (9). In contrast, short chain fatty acids (SCFAs), produced from the fermentation of dietary fibers, have demonstrated protective effects against CRC, suggesting that a high-fiber diet may mitigate cancer risk (9, 10).

Recent advancements in metagenomics and metabolomics have further elucidated these diet-microbiome-CRC links. Meta-analyses of metagenomic datasets from various geographic regions have identified globally associated CRC microbes, including *Fusobacterium nucleatum*, *Peptostreptococcus stomatis*, and *Gemella morbillorum*, among others such as *Bacteroides fragilis*, *Parvimonas* spp., *Clostridium symbiosum*, and *Solobacterium moorei* (11). These microbial shifts are associated with CRC-enriched predicted metabolites such as amino acids, cadaverine, and creatine (11), as well as secondary bile acids (12). Additionally, functional analyses have highlighted CRC-enriched pathways, such as protein and mucin catabolism, alongside a depletion in carbohydrate degradation pathways (12). These findings underscore the complex interplay between dietary factors, gut microbiota, and CRC development.

Hence, individuals at elevated risk for CRC, including those with adenomas or prior disease, may already exhibit microbial dysbiosis, mucosal alterations, or inflammatory changes associated with early carcinogenesis. These baseline differences could influence how the gut microbiome responds to dietary exposures, limiting the generalizability of findings from healthy populations. Prior systematic reviews have largely focused on diet and microbiome outcomes in general populations, diet and CRC risk associations, or observational evidence (6, 13–18, 20–22, 38) but have not specifically evaluated how diet shapes the gut microbiome within high-risk CRC groups.

This systematic review addresses this gap by synthesizing evidence from randomized controlled trials that examine dietary interventions in individuals at increased CRC risk, defined as those with adenomatous or precancerous polyps, colorectal adenomas, a personal or family history of adenomas or CRC, and those identified as high risk through clinical screening tools. In each case, CRC-risk status was designated by the original study on the basis of clinical, genetic, or family-history evidence, rather than inferred from a coexisting condition. These interventions, including specific foods, nutrients, supplements, and overall dietary patterns, are evaluated for their effects on gut microbial diversity, taxonomic composition, and metabolite profiles to clarify mechanisms through which diet may influence CRC risk. In doing so, this review aims to assess the current evidence base on diet and the gut microbiome in individuals at elevated risk for CRC and to guide future research in this area.

## Methods

The study protocol is registered with PROSPERO, the International Prospective Register of Systematic Reviews (CRD42024530038).

### Search strategy

A search strategy, developed in accordance with the Cochrane Handbook of Systematic Reviews (23), is detailed in Supplementary Table S1. The search was designed to identify dietary interventions examining the gut microbiota in individuals at risk for CRC. Primary searches were conducted on April 09, 2024, and then updated on July 28, 2025, with no restrictions being imposed on the date of publication.

### Study selection

The search was conducted in three databases (PubMed, Scopus, and Web of Sciences) and the results were compiled in Zotero (version 6.0.23). To identify additional relevant studies, reference lists of related systematic reviews and meta-analyses were manually reviewed. Three authors (JD, SP, JS) systematically assessed the articles for inclusion. Discrepancies were resolved through a voting process. Details of the PICO framework can be found in Supplementary Table S2.

The study selection process is illustrated in the PRISMA (Preferred Reporting Items for Systematic Reviews and Meta-Analyses) flow diagram (24) (Figure 1). The exclusion process was conducted in four stages. In the first pass, studies were excluded if they were duplicates, involved children, pregnant individuals, non-human subjects, or *in vitro* experiments. Reviews, study protocols, non-research articles, genome analyses, text-mining, food nutrient analysis, food quality studies, and studies where the full text or abstract was not available in English were also excluded.

**Figure 1:**
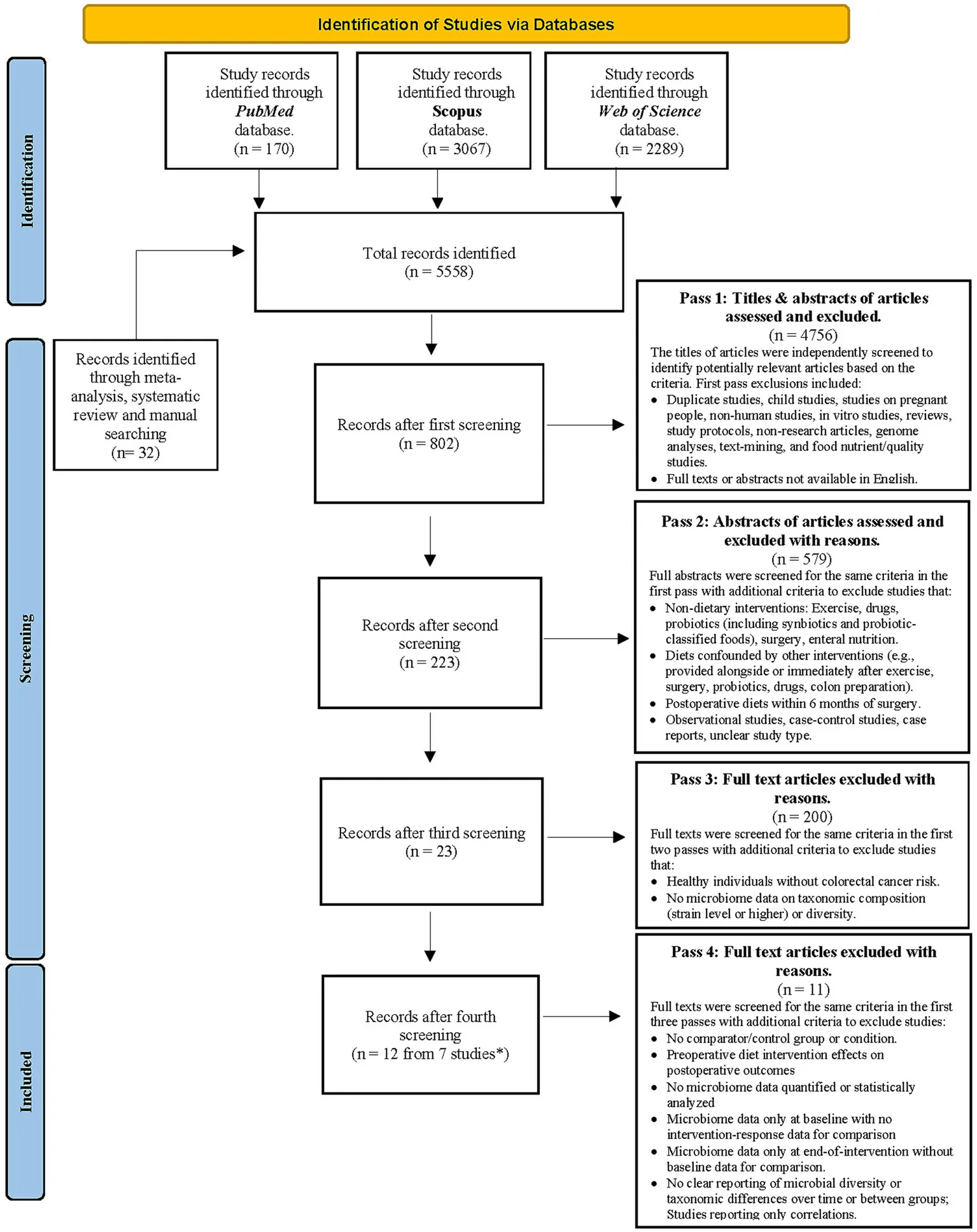
PRISMA flow diagram summarizing the study selection process (24). Two studies analyzed had multiple associated papers. *, 2 studies had multiple papers.

In the second pass, exclusions included studies focusing on interventions other than diet, such as exercise, drugs, probiotics (including foods classified as probiotics by the study), synbiotics, surgery, or enteral nutrition. Dietary supplements described as drugs or registered with the FDA or international regulatory agencies as drugs were also excluded. Studies where dietary effects could not be isolated, such as those involving diets provided alongside or immediately after exercise, surgery, probiotics, drugs, or colon preparation interventions, were excluded. Diets provided postoperatively within six months of surgery, where surgical effects could confound outcomes, were also excluded. Observational studies, case–control studies, case reports, and studies unclear about whether they were observational or dietary interventions were removed.

In the third pass, studies were excluded if the population were healthy without CRC risk or if CRC risk was classified solely based on diet. Accordingly, trials in populations defined by other disease status (obesity, type 2 diabetes, inflammatory bowel disease, etc.) were eligible only where the authors defined the population as having elevated CRC risk. Studies were also excluded if they did not provide microbiome data related to taxonomic composition (strain level or higher) or diversity. Additionally, studies presenting microbiome data only at the ASV/OTU level without aggregation to taxonomic composition were excluded.

In the fourth pass, exclusions were based on whether the study addressed the research question. Studies examining preoperative dietary interventions effects on postoperative outcomes were excluded. Studies were excluded if no microbiome data were quantified or statistically analyzed; if microbiome data were only presented at baseline with no intervention response data presented for comparison; or if microbiome data were only presented at the end of the intervention without baseline data for comparison. Additional exclusions included studies without a comparator or control group/condition, studies without clear reporting of differences in microbial diversity or taxonomic abundances over time or between groups. Studies that reported only correlations were also excluded. Papers replicating information already presented in another paper from the same study were not included in the review.

### Data extraction

Data collection tables were designed and finalized by three authors (JD, SP, JS). Data extraction was performed by SP and JS, with JD reviewing for accuracy. Study quality was assessed using the Oxford Centre for Evidence-Based Medicine Levels of Evidence (25), and risk of bias was evaluated using the Academy of Nutrition and Dietetics quality control checklists (19).

## Results

In total 5,558 records were screened for inclusion from database search and manual searching of meta-analyses, systematic reviews, and reference lists. A total of 5,546 records were excluded based on the established criteria (Figure 1). Seven studies with dietary interventions that discussed microbiome outcomes in individuals at elevated risk for CRC were included in the systematic review. Two studies had multiple publications discussing different microbiome outcomes (26–32) and are grouped together in Table 1.

**Table 1:** Characteristics of studies examining dietary interventions and gut microbiota in individuals at risk for colorectal cancer.

Study	Intervention	Duration	Participant profile	Microbiome and metabolite outcomes
Prakash et al. (34)	Ginger extract intervention (*n* = 33): 500 mg ginger extract capsule (5% gingerols)/capsule. Approx. 4 capsules/day. Control (*n* = 35): lactose capsule. Approx. 4 capsules/day.	6 wk. intervention + 6 wk. washout	68 (46 F, 22 M); mean age: 60.2 yr.; mean BMI: 28.5 kg/m2; diagnosis of colorectal adenoma within the last 5 yr	Ginger extract vs. control No differences in alpha- or beta-diversity were observed Decrease over 6 wk. in *Bacteroides*, *Akkermansia*, and *Ruminococcus*
Turunen et al. (37)	β-glucan intervention (*n* = 11): Bread (125 g/d) enriched with barley-derived β-glucan (3 g/d) Control (*n* = 9): Bread (125 g/d) without barley-derived β-glucan	12 wk	20 (10 M & 10 F); mean age: 57.6 yr.; BMI: not provided; polypectomized individuals with biopsy-confirmed adenomatous polyps ≥1 cm or ≥3 polyps with moderate/severe dysplasia.	β-glucan: Total coliform decreased over 4 wk. *Clostridium perfringens* decreased from 4 wk. to 12 wk. No significant changes in total volatile fatty acids (VFA), propionate, branched-VFA, or other-VFA concentrations. β-glucan vs. control: Higher acetate at 12 wk. Lower butyrate at baseline and 12 wk.
So et al. (36)	Rice bran intervention (*n* = 19*): 30 g per day Rice powder control (*n* = 20*): 30 g per day *completers	24 wk	40 (27 M, 13 F); mean age: 65.6 yr.; mean BMI: 24 kg/m2; high risk of CRC as assessed by the Asia-Pacific Colorectal screening tool	Rice bran vs. rice powder: Greater increase in Firmicutes and *Lactobacillus* over 24 wk. No differences in alpha-or beta- diversity
Sheflin et al. (30), Baxter et al. (26), Brown et al. (27), Zarei et al. (32)	Heat-stabilized rice bran (RB; 30 g; *n* = 9*) incorporated into one meal and snack daily Whole navy bean powder (NB; 35 g; *n* = 10*) incorporated into one meal and snack daily Control group (*n* = 10*): macronutrient- matched to intervention groups; One meal and snack daily *completers Baxter et al. (26): 8 in NB and 10 in control Brown et al. (27): 9 in RB and 10 in control Zarei et al. (32): 10 in NB and 10 in control	4 wk	37 (12 M, 17 F); mean age: 62 yr.; mean BMI: 28 kg/m2; CRC survivors more than 4 mon post cancer treatment (chemotherapy or radiation).	RB: Increase in diversity indices Chao, Sobs, Simpson at 4 wk. vs. BL (30) Increased Bacteroidetes and decreased Firmicutes, decreased (F: B ratio) at 2 wk. vs. BL (30) Significant increase in microbial metabolic pathways (e.g., phenylpropanoid biosynthesis, other glycan degradation, starch/sucrose metabolism, streptomycin biosynthesis, and sphingolipid metabolism) and decrease in biosynthesis of unsaturated fatty acids at 2 wk. vs. BL (30) Greater increase in stool propionic acids over 2 wk., and stool acetic acid over 2 wk. versus control (30) Decrease in stool N-acetylhistamine, glycodeoxycholate, p-cresol sulfate over 4 wk (27) Increase in stool beta-hydroxyisovalerate and diosmin over 4 wk. and diosmin also greater than control at 4 wk (27) No differences in other stool bile acid or polyphenol metabolites over time (30) NB: Increased stool diversity (Chao index) at 4 wk. vs. BL (30) No change in taxonomic composition or pathways over time (30) No differences in SCFA fold-change vs. control (30) Stool metabolites: Cadaverine and 4-hydroxyphenylacetate increased and caprylate decreased over 4 wk. vs. BL (26) Higher hydantoin-5 propionic acid vs. control at 4 wk (26) Plasma metabolites (32) Greater 3-(4-hydroxyphenyl)propionate and 2,3-dihydroxy-2-methylbutyrate at 4 wk. versus control 2,3-dihydroxy-2-methylbutyrate also increased over 4 wk. Urine metabolites (32) N2, N5-diacetylornithine, 4-hydroxyhippurate, and salicylate increased over 4 wk. Greater N2, N5-diacetylornithine at 4 wk. vs. control
Zhang et al. (35)	Navy beans intervention (NB): 1 cup/day of cooked navy beans Control: usual diet	8 wk. each phase; no washout	48 (25 M, 23F); mean age: 62.4 yr.; mean BMI: 30.8; history of precancerous colon/rectal polyps or CRC survivor post-treatment with maintained bowel length	NB over 8 wk.: Increased diversity (inverse Simpson index) Increased *Faecalibacterium*, *Odoribacter*, *Bifidobacterium*, and *Eubacterium brachy* Decreased *Collinsella* and *Ruminococcus torques* Increased pipecolic acid, trigonelline, and decreased indole-derivative
Djuric et al. (28) Griffin et al. (29) Umoh et al. (31)	Healthy Eating (HD, *n* = 61): ≥ 5 ½-cup servings fruits/vegetables daily (include dark green/orange), ≥3 servings whole grains, <10% calories from saturated fat Mediterranean Diet (MD, *n* = 59): 30% calories from fat (PUFA: SFA: MUFA ratio 1:2:5), omega-3 fatty acids ≥2x/wk., 7–9 ½-cup servings fruits/vegetables daily, daily herbs & alliums (29): 58 in HD and 58 in MED	26 wk	120 (34 M, 86 F); mean age: 52.5 yr.; mean BMI: 27 kg/m2; increased colon cancer risk: family history in a first-degree relative or two second-degree relatives or personal history of adenoma or colon cancer	MD: No changes in colon mucosal microbiome taxa or diversity (28) HD: Decrease in community θYC distance (dissimilarity) over 26 wk. but no changes in taxa HD and MD: No changes in TMAO (29) and LPS-binding protein (31) Decrease in total branched-chain bacterial fatty acids over 26 wk (31)
Byrd et al. (33)	High-fiber, high-fruit, high-vegetable intervention (HFV, *n* = 233): ≥ 18 g of fiber per 1,000 kcal ≥ 3.5 servings of fruits and vegetables per 1,000 kcal ≤ 20% of kcal/day of fat Individualized goals set by dietitian Control (*n* = 222): general dietary guidelines from the National Dairy Council	208 wk	455 (317 M, 138 F); mean age: 61.6 years; mean BMI: 27.8 kg/m^2^; history of colorectal adenomatous polyp removal 6 mon prior to starting study	HFV vs. Control: Greater decrease in genera Porphyromonas and Prevotella over 208 wk. No changes in diversity

^1^CRC, Colorectal Cancer; BMI, Body mass index; wk, Week.

### Study design and duration

Five studies were conducted in the United States (28, 30, 33–35), 1 in China (36), 1 in Greece (37). Included studies varied in design and intervention duration tested (Table 1). Six studies (26–34, 36, 37) used parallel-arm randomized controlled trial (RCT) designs while one study (35) employed a randomized crossover design. Study durations ranged from 4 wk to 208 wk.

### Dietary interventions

The interventions studied were diverse ranging from dietary supplements such as ginger extract (34), functional foods such as rice bran (36) and β-glucan-enriched bread (37), whole foods such as navy beans (35), enriched meals and snacks containing rice bran or whole navy bean powder (30), and dietary patterns such as healthy eating or Mediterranean dietary patterns (28) and those that emphasize fruits, vegetables, and fiber (33).

### Population characteristics

The studies examined diverse populations with varying CRC risks and treatment histories. Participants included individuals with current or recently removed adenomatous polyps (33, 37), colorectal adenomas (34), and precancerous colon or rectal polyps (35). Other studies defined risk based on clinical tools such as the Asia-Pacific Colorectal Screening Tool (36) or family or personal history of adenomas or colon cancer (28, 29, 31). Several studies included CRC survivors more than four months post-treatment for chemotherapy or radiation (26, 27, 30, 32). Participants were predominantly middle-aged to older adults with mean ages ranging from the 50s to 70s and mean BMIs in the overweight and obesity range.

### Effects of dietary intervention on microbial diversity

Navy bean consumption increased stool microbial diversity, with significant increases in diversity indices such as the inverse Simpson index over 8 wk (35) and Chao index over 4 wk (30). Similarly, rice bran interventions showed an increase in Chao, Sobs, and Simpson diversity indices over 4 wk (30), but no differences in diversity over 24 wk (36). Dietary patterns such as Healthy Eating and Mediterranean diets had no significant impact on colon mucosal microbiota diversity. However, a decrease in community θYC distance, indicating reduced dissimilarity, was observed in the Healthy Eating group over 26 wk (28). A high-fiber, high-fruit, high-vegetable, low-fat dietary intervention showed no significant changes in rectal tissue microbiota alpha or beta diversity (33). Ginger extract supplement did not result in significant differences in alpha or beta diversity (34). The β-glucan intervention did not assess diversity indices (37).

### Effects of dietary intervention on microbial taxonomic composition

Navy bean consumption over 8 wk resulted in increased abundance of *Faecalibacterium*, *Odoribacter*, *Bifidobacterium*, and *Eubacterium brachy*, with decreases in *Collinsella* and *Ruminococcus torques* (35). However, in another intervention, whole navy bean powder showed no changes in taxonomic composition over 4 wk (30). In the same study, rice bran increased the abundance of Bacteroidetes while decreasing Firmicutes, leading to a reduction in the Firmicutes-to-Bacteroidetes ratio at 2 wk compared to baseline (30). In a 24 wk duration rice bran intervention, a greater increase in Firmicutes and *Lactobacillus* compared to rice powder was observed (36). The ginger extract intervention resulted in significant decreases in *Bacteroides*, *Akkermansia*, and *Ruminococcus* compared to placebo over 6 wk (34). The β-glucan intervention decreased total coliform abundance over 4 wk and *Clostridium perfringens* abundance from 4 to 12 wk (37). No significant changes in taxonomic composition were observed with Healthy Eating or Mediterranean diets in colon mucosal microbiota (28). A high-fiber, high-fruit, high-vegetable, low-fat dietary intervention showed significant reduction in the relative abundances of *Porphyromonas* and *Prevotella* in the rectal tissue microbiota over 208 wk.

### Effects of dietary intervention on microbial-derived metabolites

Navy bean consumption over 8 wk increased pipecolic acid and trigonelline while decreasing an indole derivative (not specified) (35). In the other navy bean intervention, stool cadaverine and 4-hydroxyphenylacetate increased over 4 wk (26). Additionally plasma 3-(4-hydroxyphenyl)propionate and urinary 4-hydroxyhippurate was higher at 4 wk compared to control (32). In the same study, rice bran increased stool acetic and propionic acids over 2 wk compared to control (30) and decreased stool glycodeoxycholate, and p-cresol sulfate over 4 wk (27). Additional increases in stool diosmin were observed over 4 wk (27). The β-glucan intervention showed no significant changes in total volatile fatty acids (VFA), propionate, branched VFAs, or other-VFA concentrations. However, higher acetate concentrations were observed at 12 wk compared to placebo (37). A decrease in total branched-chain bacterial fatty acids (31) but no changes in trimethylamine-N-oxide (TMAO) (29) or lipopolysaccharide-binding protein (LPS-binding protein) (31) were observed in Healthy Eating and Mediterranean diets over 26 wk. Supplementary Table S3 lists all metabolites and compounds reported across these trials, classified as microbially-derived or of unclear origin.

### Quality of assessment of studies

These sources of methodological heterogeneity may partially account for inconsistencies in reported diversity, taxonomic and metabolite outcomes. To facilitate comparison, Supplementary Table S4 summarizes key technical features across included studies, including sample collection procedures, biospecimen processing and DNA extraction methods, sequencing or profiling platforms, bioinformatic pipelines, microbiome and metabolite outcomes assessed, outcome classification approaches, statistical analyses performed, and use of false discovery rate or other multiple-testing corrections. Standardizing microbiome sampling, sequencing, and analytical methods across future trials would improve cross-study comparability and reduce this heterogeneity.

The characteristics of the included studies are summarized in Table 2. Assessment was done on all 7 studies including the multiple publications from 2 studies (26, 27, 29, 31, 32). According to the Oxford Centre for Evidence-Based Medicine 2009 levels of evidence (25) using the Therapy/Prevention, Aetiology/Harm hierarchy, 3 studies were classified as 1b (34–36), indicating high-quality individual RCTs, while 4 studies was classified as 2b (26–33, 37), indicating studies with less than 80% follow-up, participant retention, or participant data analyzed.

**Table 2:** Risk-of-bias analysis of studies examining dietary interventions and gut microbiota in individuals at risk for colorectal cancer.

Study	Selection of participants free from bias?	Study with >80% follow up or participant data analyzed^2^	Standard/valid/reliable data collection procedures	QCC rating^3^	Evidence grade
Prakash et al. (34)	Yes	Yes	Yes	+	1b
Turunen et al. (37)	Unclear	No	Yes	-	2b
So et al. (36)	Yes	Yes	Yes	+	1b
Sheflin et al. (30)	Yes	No	Yes	∅	2b
Baxter et al. (26)	Yes	No	Yes	∅	2b
Brown et al. (27)	Yes	No	Yes	∅	2b
Zarei et al. (32)	Yes	No	Yes	∅	2b
Zhang et al. (35)	Yes	Yes	Yes	+	1b
Djuric et al. (28)	Yes	No	Yes	∅	2b
Griffin et al. (29)	Yes	No	Yes	∅	2b
Umoh et al. (31)	Yes	No	Yes	∅	2b
Byrd et al. (33)	Yes	No	Yes	∅	2b

^1^QCC, Quality Criteria Checklist.

^2^Follow-up here takes into account participant attrition or participants included in analysis.

^3^QCC rating (19): +, report has clearly addressed issues of inclusion/exclusion, bias, generalizability, and data collection and analysis; ∅, report is neither exceptionally strong nor exceptionally weak; −, report has not adequately addressed issues of inclusion/exclusion, bias, generalizability, and data collection and analysis.

^4^Oxford Centre for Evidence-Based Medicine Levels of Evidence 1 (25).

Using the Academy of Nutrition and Dietetics Quality Criteria Checklist for Primary Research risk-of-bias assessment (19), 3 articles (34–36) were rated positively (+), reflecting adequate consideration of bias, participant retention, and robustness, validity, and rigor in the intervention design, data collection, and analysis. Eight articles (26–33) were rated neutral (∅), indicating they were neither exceptionally robust nor notably weak. One article (37) was rated negative, primarily because it was a pilot study that did not adequately address all validity criteria. Most studies in this review exhibited statistical limitations, particularly in assessing group-by-time interactions to analyze microbiome outcomes comprehensively. Common issues included reliance on within-group changes without assessing intergroup differences over time in the same statistical model. Other issues pertained to lack of clarity in *p*-value reporting, and inconsistent application of false discovery rate (FDR) corrections for taxa-level analyses.

## Discussion

### Summary of evidence

This systematic review highlights the limited availability of RCT dietary interventions focusing on microbiome outcomes in individuals at elevated risk for CRC. Earlier evidence largely stems from observational studies examining associations between microbiome profiles and CRC risk, or diet and CRC risk (6, 13–18, 20–22, 38). Additionally, many dietary intervention trials that were excluded focused on pre- or post-operative microbiome outcomes, recovery from surgery, or dietary effects combined with drugs or other therapies. Such studies, while valuable, do not provide direct insights into the potential of dietary interventions for modulating the microbiome for CRC prevention or recurrence in elevated-risk populations. In this context, the findings of this review shed light on how specific dietary interventions influence microbial diversity, taxonomic composition, and microbial-produced metabolites in CRC-risk individuals.

### Microbial diversity and taxonomic composition shifts

Across interventions, effects on microbial diversity metrics were minimal and were observed only with the HD diet (28), navy bean powder interventions (30, 35), and with one rice bran intervention (30). No consistent patterns emerged by intervention type (functional compounds, whole foods, or dietary pattern), intervention duration, or by diversity metric. Although the number of available RCTs in CRC-risk populations remains small, the current evidence may suggest that diversity metrics are less responsive to dietary effects within this population.

In contrast, taxonomic changes, although directionally mixed, were more consistently observed at finer taxonomic resolution, particularly at the genus level, and were context dependent. In some interventions (rice bran, navy bean), enrichment of taxa commonly described as beneficial, including Faecalibacterium, Bifidobacterium, and Lactobacillus, was observed (35, 36). These are taxa frequently discussed in relation to butyrate production, anti-inflammatory properties, and epithelial homeostasis (39–44). Conversely, other interventions [ginger extract (34)] were associated with reductions in taxa including Akkermansia and Ruminococcus, genera that include species involved in mucin degradation and complex carbohydrate metabolism (45–47).

Fluctuating shifts were also observed in the pathogenic *Clostridium perfringens* (beta-glucan–enriched) (37, 48); decreases in Porphyromonas (HFV diet (33)), a genus in which certain species have been reported as differentially abundant in CRC cohorts (12); and changes in Bacteroides (ginger extract) and Prevotella (HFV diet), genera with heterogeneous species-level functions and context-dependent associations with host health (49, 50). These findings underscore that increases or decreases in specific taxa cannot be interpreted uniformly without consideration of species-level heterogeneity and ecological context.

Additionally, taxonomic differences observed between navy beans and navy bean powder (30, 35) suggest that food matrix and processing may influence microbial accessibility to substrates. Processing, such as grinding whole foods into powder form, may alter nutrient bioavailability (51, 52) and microbial utilization patterns.

Overall, whether such taxonomic shifts represent substrate-driven microbial competition or changes with meaningful implications for host health remains uncertain. Collectively, these findings indicate that directionality alone (increase or decrease) does not uniformly signify benefit or harm, and food form must be considered when interpreting compositional shifts. Building on these structural changes in the microbiota, an examination of microbial metabolites shifts provides critical insights into the functional impacts of dietary interventions and their relevance to CRC prevention.

### Microbial produced metabolites

Among the four studies reporting metabolite outcomes, increases in short-chain fatty acids and polyphenol- and plant-derived metabolites related to gut barrier support and anti-inflammatory and antioxidant processes were reported in the studies that assessed these metabolites (53–64). Concurrent decreases were observed in secondary bile acids, p-cresol sulfate, and branched-chain bacterial fatty acids (27, 65–70). Where reported, these changes may suggest suppression of tumor-promoting pathways, proteolytic fermentation or altered substrate utilization. Figure 2 illustrates the effects of dietary interventions on microbially-derived and other metabolites, with each compound’s classification and potential implications for CRC risk detailed in Supplementary Table S3 (87–120).

**Figure 2:**
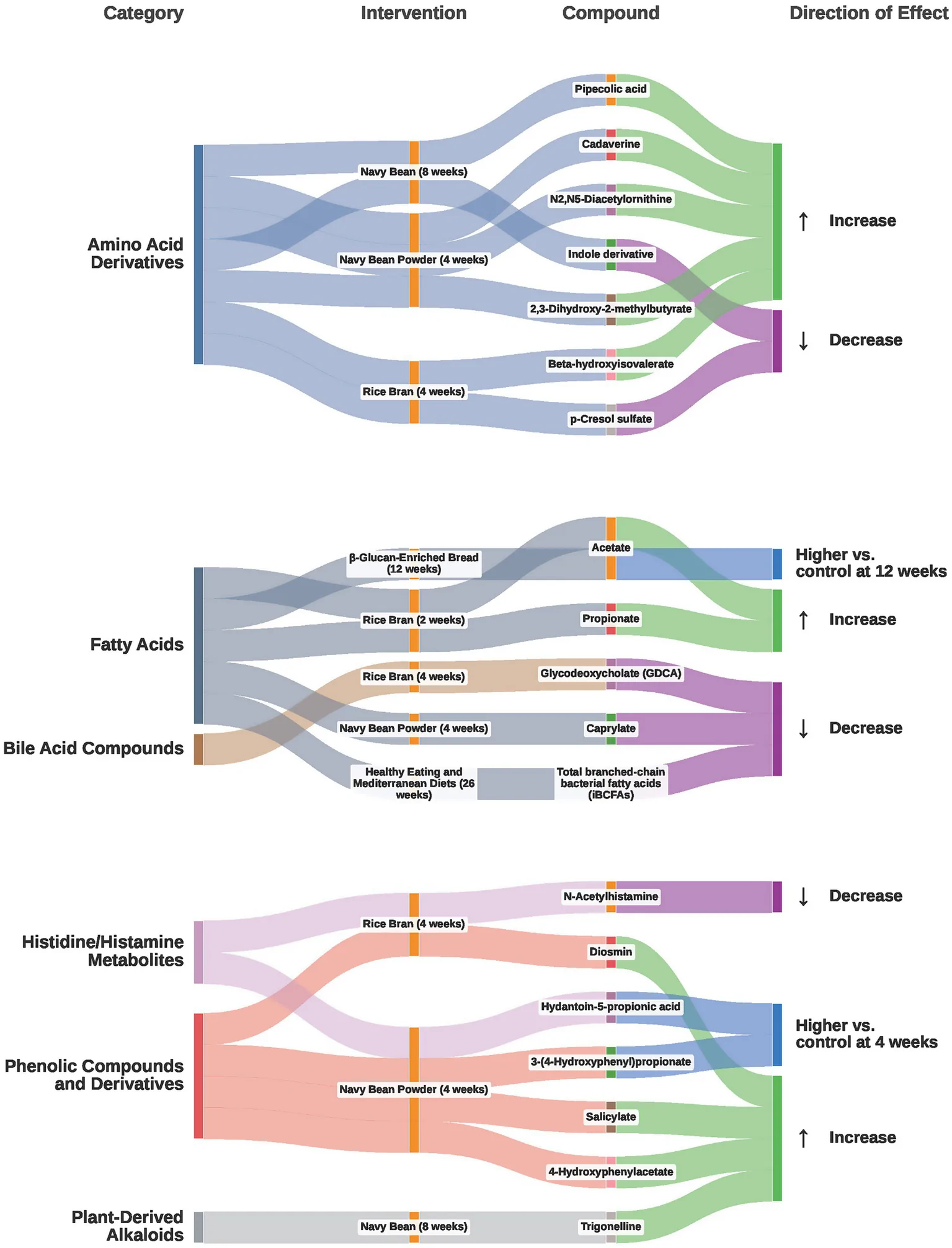
Sankey diagram illustrating the effects of dietary interventions on microbially-derived and other metabolites in individuals at elevated risk for CRC. The first column categorizes metabolites based on their biochemical or functional classification, and each class is assigned a unique color to indicate metabolite type. The second column lists the dietary interventions, and the color serves as a linking factor between interventions and metabolites. The third column displays the specific metabolites affected by these interventions. The final column indicates how each metabolite responded, with the color of the flow into this column denoting the type of result: green denotes a within-group increase and purple a within-group decrease relative to baseline over the intervention period, while blue denotes a metabolite that was higher versus the control at the indicated timepoint.

Although most trials did not evaluate direct clinical endpoints such as adenoma recurrence or mucosal biomarkers, the directionality of metabolite changes observed across studies is consistent with pathways linked to epithelial proliferation, inflammatory signaling, and tumor-promoting metabolic environments, supporting biological plausibility for CRC risk modification.

The observed metabolite shifts underscore the microbiome’s critical role as a mediator between diet and host physiology, influencing CRC risk. Microbial mechanisms contributing to CRC risk include the “driver-passenger” model theory which suggests that “driver” bacteria, initiate CRC by causing DNA damage, leading to the accumulation of mutations in colonic epithelial cells (20, 71). These driver bacteria are eventually replaced by “passenger” bacteria, which thrive in the altered tumor microenvironment both promoting inflammation through activation of pathways such as NF-κB and Wnt/β-catenin, and tumor progression (20, 71). Another theory purports that microbial dysbiosis can alter host energy metabolism by shifting from oxidative phosphorylation to glycolysis (Warburg effect), downregulating the tricarboxylic acid (TCA) cycle, and enhancing lactate production, hallmarks of cancer metabolism (22). Dietary interventions can modulate these microbiome-mediated pathways to reduce CRC risk. *In vitro* studies demonstrate that SCFAs can inhibit histone deacetylase (HDAC) activity, supporting tumor suppression pathways (72, 73). Resistant starch, which is readily metabolized by bacteria, and found in many foods, can influence CRC-related DNA methylation patterns, enhancing genome stability (e.g., GADD45A upregulation), decreasing cell cycle proliferation (e.g., CDK4 expression), and lowering crypt mitotic cell proportion in the upper half of the crypt in tumor tissue (13, 74), contributing to anti-neoplastic effects. Additionally, polyphenols, either directly or through their microbial degradation byproducts, can reduce CRC risk by exerting antioxidant and anti-inflammatory effects, modulating signaling pathways, enhancing gut barrier integrity, promoting butyrate production, exhibiting selective antibacterial activity, and suppressing carcinogenic bacteria (18). This highlights the potential of dietary interventions to modulate these complex microbiome-mediated mechanisms.

### Strengths, limitations, and interpretation considerations

This systematic review’s strength lies in its exclusive focus on RCTs, providing a stronger quality of evidence on how dietary interventions influence the gut microbiome in individuals at elevated risk for CRC. Many existing studies on diet and CRC have been prospective cohort or case–control studies, particularly in the context of microbiome outcomes. While such studies have been included in previous reviews (6, 13–18, 20–22, 38), this review emphasizes the scarcity of RCTs, underscoring the need for more controlled studies, particularly in diverse populations. The review also provides a detailed analysis of microbial-derived metabolites, offering mechanistic insights into how dietary interventions may modulate CRC risk through metabolic pathways.

A limitation of this review is the inability to explore differences across the lifespan. Age-related changes in the gut microbiome such as reduced diversity and differences in taxonomic composition and functional capacity (75, 76), may influence how individuals respond to dietary interventions. While older adults have traditionally been at higher CRC risk, recent trends indicate a plateau in this age group, whereas early-onset CRC is rising among younger adults (<50 years) (77, 78). Moreover, while nutrient-rich dietary patterns consumed earlier in life may attenuate this risk (79, 80), the lack of age-stratified RCTs prevented an in-depth analysis of these potentially critical life-stage differences. The magnitude and direction of the microbiome changes are likely contingent on baseline host–microbial ecology. Individuals with established CRC or recent treatment exposure may exhibit altered microbial structure, epithelial integrity, immune activation, and metabolic signaling compared with high-risk but otherwise disease-free individuals, potentially modifying dietary responsiveness. Moreover, included trials sampled either stool or mucosal tissue, so effects specific to the luminal versus mucosal microbiota could not be distinguished. Additionally, studies primarily focused on short- to medium-term dietary interventions, limiting insights into the long-term effects of diet on the microbiome and CRC risk. This constraint underscores the need for cautious interpretation of the findings. Moreover, the limited number of trials and heterogeneity of outcomes also precluded quantitative pooling of specific outcomes via-meta-analyses, as a result the evidence was synthesized narratively.

### Implications of findings, research gaps, and future directions

A critical gap highlighted by this systematic review is the limited availability of RCTs examining the effects of dietary interventions on gut microbiome outcomes in individuals at elevated risk for CRC. While the reviewed studies provide valuable insights into how specific foods and dietary patterns influence microbial diversity, taxonomic composition, and metabolite production, translating these findings into clinical strategies for CRC prevention would require more trials and subsequently meta-analyses of these outcomes.

Future studies should also move beyond cataloging microbial composition and focus on how microbial functions influence CRC-related pathways, including inflammation, epithelial integrity, and immune modulation. One notable research gap is the underutilization of advanced methodologies to assess the functional capacity of the microbiome (81). While many studies have measured common microbial metabolites such as SCFAs and secondary bile acids, few have employed advanced functional analyses, meta-transcriptomics or metabolomics, to elucidate other microbial pathways involved in amino acid metabolism, polyphenol biotransformation, generation of bioactive metabolites, and their influence on host metabolic and immune responses. Incorporating these analyses into future dietary interventions could uncover novel mechanisms by which diet influences CRC risk through microbiome modulation. Future trials should more explicitly focus on microbial-derived metabolites that are directly implicated in CRC-relevant pathways, including DNA damage, epithelial proliferation, inflammatory signaling, and tumor metabolism, to strengthen clinical interpretability.

The findings also underscore the need to examine whole dietary patterns rather than isolated food components. Much of the existing literature focuses on selected dietary components, such as fiber, without considering the broader dietary context that accounts for macronutrient and micronutrient interactions. For example, while fiber is widely recognized for its gut health benefits (82, 83), its effects on the microbiome may be more pronounced within nutrient-rich diets that contain other healthy dietary components such as polyphenols (84). Similarly, fiber intake may counteract some of the adverse effects of high red meat consumption on CRC risk and inflammatory outcomes (17, 85). It may not be individual dietary components alone but rather the overall dietary pattern that influences CRC risk. However, this nuanced approach, considering dietary patterns in RCTs to assess microbiome-related outcomes, remains underexplored in CRC prevention research.

Additionally, most studies have predominantly focused on Western diets and populations limiting the broader applicability of the findings. Expanding RCT research to include dietary patterns predominant in regions with low CRC risk such as Southern Asia, or Sub-Saharan Africa (86), could provide valuable insights into protective dietary components and distinct diet-microbiome interactions. This approach could help identify how interaction of specific dietary elements (e.g., high fiber content, unique polyphenols, or fermented foods) contributes to the reduced CRC risk observed in these populations. Formal meta-analyses could also compare dietary microbiome responses between elevated-risk and average-risk populations to determine whether intervention effects generalize across baseline risk states.

To advance this field, future studies should prioritize robust trial designs with clear endpoints that also extend beyond microbiome outcomes to include host-relevant outcomes, such as gut barrier function, systemic inflammation, and specific cancer-related pathways. Utilizing insights from epidemiological and computational studies that highlight CRC-associated metabolic signatures could guide the testing of dietary interventions associated with specific microbial or metabolic pathways. Addressing these research gaps could pave the way for microbiome-targeted dietary strategies that are not only preventive but also tailored to individual risk profiles, potentially transforming CRC prevention and management strategies.

## Conclusion

This systematic review emphasizes the significant yet underexplored potential of dietary interventions to modulate gut microbiome pathways relevant to CRC prevention. While current evidence demonstrates that whole foods, nutrient-rich dietary patterns, and specific functional compounds can positively influence microbial diversity, composition, and metabolite production, substantial research gaps remain. Future studies must prioritize RCTs on dietary patterns in diverse populations and integrate advanced microbiome functional analyses to better delineate the diet-microbiome-CRC axis. As the evidence base expands, future reviews should synthesize comparable outcomes through meta-analyses and formally rate the certainty of evidence before it can be applied for tailoring dietary strategies to effectively reduce CRC risk through microbiome modulation.

## Data Availability

The original contributions presented in the study are included in the article/Supplementary material, further inquiries can be directed to the corresponding author.

## Supplementary material

SUPPLEMENTARY TABLE S1 Details of search methods.

SUPPLEMENTARY TABLE S2 PICO framework guiding the systematic review.

SUPPLEMENTARY TABLE S3 Classification of microbially-derived and other metabolites by biochemical origin and their potential implications for CRC risk.

SUPPLEMENTARY TABLE S4 Study-level methodological details for microbiome and metabolite analyses in included randomized controlled trials.
