# Supplementary material for: A systematic review of the effects of diet on the gut microbiota in individuals at elevated risk for colorectal cancer
Source: Front Nutr. 2026 Oct 16;13:1818522. doi: 10.3389/fnut.2026.1818522 (PMC13719305; doi:10.3389/fnut.2026.1818522)
Supplement: Supplementary file 1 [file Table_1.DOCX]

**Supplementary Table S1**. Details of search methods

| **Database** | **Web Address** | **Keywords** |
| --- | --- | --- |
| PubMed | <http://www.ncbi.nlm.nih.gov/pubmed/> | (Diet OR nutrition OR nutrients OR food OR Supplement) AND (colorectal OR colon OR rectal) AND (cancer OR neoplasm OR adenocarcinoma OR metastasis OR tumor) AND (microbiome OR microbiota OR microflora OR microbiomes OR microbe OR microorganism OR microbiology OR microbial OR flora OR bacteria) |
| Web of Science | <https://www.webofscience.com/> | Topic -- ( diet OR nutrition OR nutrients OR food OR supplement ) AND ( colorectal OR colon OR rectal) AND (cancer OR neoplasm OR adenocarcinoma OR metastasis OR tumor)AND microbiome OR microbiota OR microflora OR microbiomes OR microbe OR microorganism OR microbiology OR microbial OR flora OR bacteria ) |
| SCOPUS | <https://www.scopus.com/> | TITLE-ABS-KEY ( ( ( diet OR nutrition OR nutrients OR food OR supplement ) AND ( colorectal OR colon OR rectal ) AND ( cancer OR neoplasm OR adenocarcinoma OR metastasis OR tumor ) AND ( microbiome OR microbiota OR microflora OR microbiomes OR microbe OR microorganism OR microbiology OR microbial OR flora OR bacteria ) ) ) |

The databases (PubMed, Web of Science, and Scopus) were searched on April 09, 2024 and then updated on July 28, 2025 by JD, SP, and JS. Following the final searches, the protocol was registered. Articles were reviewed and screened by three authors (JD, SP, JS) based on established criteria. All search terms were free-text keywords combined with Boolean operators (AND, OR) across four concept blocks (diet, colorectal, cancer, and microbiome). Controlled vocabulary terms for PubMed (e.g., MeSH) were not applied. The deliberately broad free-text strings retrieve the records that controlled-vocabulary indexing would return, together with recent or not-yet-indexed records.

**Supplementary Table S2**. PICO framework guiding the systematic review

| **Category** | **Inclusion Criteria** | **Exclusion Criteria** |
| --- | --- | --- |
| **Population** | Human adult populations (over 18 years old); individuals at elevated risk of colorectal cancer. | Children (<18 years); pregnant populations; non-human studies; studies that classify CRC risk solely based on dietary history without clinical, genetic evidence, or family history. |
| **Intervention** | Dietary interventions including dietary patterns, specific food-based or nutrient-based components, or dietary supplements. | Non-dietary interventions: Exercise, drugs, probiotics (including synbiotics and foods classified as probiotics), surgery, or enteral nutrition  Diet effects cannot be isolated: diets provided alongside or immediately after exercise, surgery, probiotics, drugs, or colon preparation interventions  Diets initiated within 6 months after surgery where surgical recovery confounds dietary effects. |
| **Comparison** | Controls, placebos, or other dietary intervention groups. | No comparator/control groups |
| **Outcomes** | Primary outcomes: microbial diversity (alpha, beta), taxonomic composition (strain level or higher). | Studies that provide only ASVs or OTUs without aggregating taxonomic composition; no clear reporting of differences in microbial diversity or taxonomic abundances over time or between groups. |

**Supplementary Table S3**: Classification of microbially-derived and other metabolites by biochemical origin and their potential implications for CRC risk

| **Compound Category** | **Intervention** | **Metabolite** | **Derivation** | **Change Observed** | **Potential Role and CRC-Relevant Implications of Metabolite** |
| --- | --- | --- | --- | --- | --- |
| **Amino Acid Derivatives** | Navy bean (8 wk ) (Zhang et al., 2023) | Pipecolic acid  *Lysine catabolite* (Zhang et al., 2023) | Microbially-derived | ↑ | Supports anti-inflammatory responses, anti-tumor and antibiotic properties potentially (Perera et al., 2015; Zarei et al., 2021a; Zhang et al., 2023). |
|  | Navy bean (8 wk ) (Zhang et al., 2023) | Indole derivative  *Byproduct of aromatic amino acid metabolism* (Liu et al., 2020) | Microbially-derived | ↓ | Indole derivatives have been found to enhance gut epithelial barrier function, activate anti-inflammatory IL-10 signaling pathways, and suppress experimental colitis (Wlodarska et al., 2017; Alexeev et al., 2018; Aoki et al., 2018; Liu et al., 2020)  CRC implications not clear. |
|  | Navy bean powder (4 wk ) (Baxter et al., 2018) | Cadaverine  *Biogenic amine* | Microbially-derived | ↑ | Involved in gut barrier function and epithelial cell turnover (Coni et al., 2019; Hanus et al., 2021).  Higher cadaverine levels observed in CRC vs. healthy controls; potential biomarker for CRC differentiation pending broader validation (Yang et al., 2019). |
|  | Navy bean powder (4 wk ) (Zarei et al., 2021a) | N2,N5-diacetylornithine  *Byproduct of urea cycle* (Razavi et al., 2020; Tang et al., 2024) | Microbial origin not clear | ↑ | Associated with diastolic dysfunction, chronic kidney disease (CKD), and sudden cardiac arrest (Luo et al., 2020; Razavi et al., 2020; Tang et al., 2024).  CRC implications not clear. |
|  | Navy bean powder (4 wk ) (Zarei et al., 2021a) | 2,3-dihydroxy-2-methylbutyrate  *Byproduct of branched chain amino acid metabolism* (Hill et al., 2023) | Microbial origin not clear | ↑ | Role and CRC implications not clear. |
|  | Rice bran (4 wk ) (Brown et al., 2017) | Beta-hydroxyisovalerate  *Byproduct of leucine metabolism* (Lee et al., 2015) | Microbially-derived | ↑ | CRC implications are unclear. |
|  | Rice bran (4 wk ) (Brown et al., 2017) | p-cresol sulfate  *Byproduct of aromatic amino acid metabolism* (Liu et al., 2020) | Microbially-derived | ↓ | P-cresol induces DNA damage in colonocytes in vitro (Al Hinai et al., 2019). |
| **Bile Acid Compounds** | Rice bran (4 wk ) (Brown et al., 2017) | Glycodeoxycholate (GDCA)  *Secondary bile acid* (Brown et al., 2017) | Microbially-derived | ↓ | Promotes tumorigenesis in vivo and inflammation in vitro in cholangiocarcinoma  (Dai et al., 2013) .  Enhanced both proliferation and migration of CRC cells in vitro (Yuan et al., 2022)**.**  Higher GDCA concentrations in CRC patients versus controls, with strong diagnostic performance for distinguishing CRC status (Yuan et al., 2022) |
| **Fatty Acids** | β-glucan-enriched bread (12 wk) (Turunen et al., 2011)  Rice bran (2 wk) (Sheflin et al., 2017) | Acetate (Turunen et al., 2011; Sheflin et al., 2017)  Propionate (Sheflin et al., 2017)  *Short chain fatty acids* | Microbially-derived | ↑ | Strengthens gut barrier integrity, reduces inflammation, supports epithelial repair (Liu et al., 2021).  Induce apoptosis in colon cancer cell lines (Hague et al., 1995) . |
|  | Navy bean powder (4 wk ) (Baxter et al., 2018) | Caprylate  *Medium-chain fatty acid* (Nagao and Yanagita, 2010) | Microbial origin not clear | ↓ | Reduced cancer cell viability and inhibited CRC cell proliferation in a dose-dependent manner, accompanied by upregulation of apoptosis-related genes and downregulation of cell cycle regulatory genes (Narayanan et al., 2015).  Lower circulating levels of caprylic acid (octanoic acid) were associated with better CRC prognosis compared to higher levels . |
|  | Healthy Eating and Mediterranean Diets (26 wk ) (Umoh et al., 2016) | Total branched-chain bacterial fatty acids (iBCFAs)  *Byproducts of protein fermentation* (Blachier et al., 2007) | Microbially-derived | ↓ | Protective role in gut inflammation (Sheflin et al., 2017; Nikolaki et al., 2023).  Although higher concentrations observed in CRC cohorts compared to controls likely owing to microbial fermentation of host-derived substrates such as sloughed intestinal cells (Gall et al., 2018) |
| **Histidine/Histamine Metabolites** | Rice bran (4 wk ) (Brown et al., 2017) | N-acetylhistamine  *Byproduct of histamine metabolism* (Weber et al., 2023) | Microbial origin not clear | ↓ | Stimulates gastric secretion (Acuña et al., 2022; Weber et al., 2023).  CRC implications not clear. |
|  | Navy bean powder (4 wk ) (Baxter et al., 2018) | Hydantoin-5-propionic acid    *Linked to histidine metabolism* (Duran et al., 1986; KEGG COMPOUND: C05565, n.d.; MetaCyc L-histidine degradation VI, n.d.) | Microbial origin not clear | ↑ | Role and CRC implications not clear. |
| **Phenolic Compounds and Derivatives** | Navy bean powder (4 wk ) (Baxter et al., 2018) | 4-hydroxyphenylacetate  *Phenolic compound* (Khanal et al., 2014) | Microbially-derived | ↑ | Potential anti-inflammatory and anti-oxidant properties (Liu et al., 2014; Zhao et al., 2018).  Specific role in CRC implications not clear. |
|  | Navy bean powder (4 wk ) (Zarei et al., 2021a) | 3-(4-hydroxyphenyl)propionate  *Proanthocyanidin metabolite* (Ward et al., 2004) | Microbially-derived | ↑ | Counters oxidative stress and inflammation (Zhang et al., 2018; Kucukgul and Nita, 2024).  Role in CRC implications is unclear. |
|  | Navy bean powder (4 wk ) (Zarei et al., 2021) | Salicylate  *Plant-derived phenolic derivative* (Hayat et al., 2007) | Microbial origin not clear | ↑ | Associated with anti-inflammatory and anti-cancer effects (Shirakawa et al., 2016).  At pharmacological concentrations in vitro shown to reduce angiogenesis triggered by colon cancer cells (Shtivelband et al., 2003). |
|  | Rice bran (4 wk ) (Brown et al., 2017) | Diosmin  *Flavonoid* (Huwait and Mobashir, 2022) | Microbially-derived | ↑ | Exhibit anti-oxidant and anti-cancer properties (Huwait and Mobashir, 2022).  Anti-proliferative effects in human colon cancer cell lines and chemopreventive activity against colon carcinogenesis in animal models (Tanaka et al., 1997; Kuntz et al., 1999). |
| **Plant-Derived Alkaloids** | Navy bean (8 wk ) (Zhang et al., 2023) | Trigonelline  *Plant alkaloid* (Zhou et al., 2012; Zia et al., 2001) | Microbially-derived | ↑ | Supports antioxidant and anti-inflammatory properties (Nguyen et al., 2024).  May reduce CRC risk by suppressing Nrf2 activation, potentially limiting oxidative stress and tumor progression (Boettler et al., 2011; Panieri et al., 2020). |

Metabolites/compounds may fall into overlapping categories; categories were selected based on biochemical context and relevance to the reviewed interventions. Derivation key: microbially-derived metabolites are those whose formation requires one or more enzymatic steps by the gut microbiota, whether through direct microbial biosynthesis, microbial biotransformation of a dietary precursor, or sequential microbial and host metabolism. Metabolites classified as unclear origin were detected following dietary intervention but lack established gut-microbial production, having been classified by the source trial within a host metabolic pathway or possibly diet-related.

**Supplementary Table S4**: Study-level methodological details for microbiome and metabolite analyses in included randomized controlled trials.

| Study | Category | Description |
| --- | --- | --- |
| (Prakash et al., 2024) | Sample Collection | Self-collected stool samples in 90% ethanol kits; transported on ice ≤72h; stored −80°C; batch processed. |
|  | Biospecimen Processing / Extraction | DNA extracted using PowerSoil Pro DNA Isolation Kit (MoBio/Qiagen). |
|  | Sequencing / Profiling Platform | 16S rRNA gene sequencing; V4 region (515F–806R); 2×300 bp paired-end; Illumina MiSeq; sequencing performed by UMN Genomics Center; negative controls included. |
|  | Bioinformatic Processing | Mothur v1.41.1; alignment to SILVA v138; chimera removal via UCHIME; OTU clustering at 99% similarity; taxonomy via RDP v18; rarefied to 8,800 reads per sample. |
|  | Microbiome / Molecular Outcomes Assessed | Alpha diversity (Shannon, Chao1); beta diversity (Bray–Curtis; ANOSIM; PCoA); longitudinal analysis via SplinectomeR; relative abundance of pre-specified CRC-associated taxa. |
|  | Outcome Classification | Microbiome outcomes = prespecified primary outcome per ClinicalTrials.gov registration (NCT03268655). |
|  | Statistical Analysis | Mixed-effects logistic regression (random intercept for subject) for taxa-level relative abundance; linear mixed-effects models for biomarker analyses; interaction term (visit×treatment) primary; α=0.05; no multiple testing correction. |
|  | FDR / Multiple Testing Correction | None; no adjustment for multiple comparisons. |
| (Turunen et al., 2011) | Sample Collection | Stool collection procedures referenced to Mitsou et al. 2010; ~4 g feces transported under anaerobic conditions (GTN anaer system) to the laboratory within ~1 hour |
|  | Biospecimen Processing / Extraction | Culture-based microbial enumeration as previously described by Mitsou et al. 2010; no DNA extraction or sequencing performed . |
|  | Sequencing / Profiling Platform | Not applicable (culture-based microbiology). |
|  | Bioinformatic Processing | Not applicable. |
|  | Microbiome / Molecular Outcomes Assessed | Viable counts (log10 CFU/g) of total aerobes, total anaerobes, coliforms, E. coli, enterococci, Bacteroides spp., Clostridium perfringens, bifidobacteria, lactobacilli, Candida spp.; fecal SCFAs by capillary GC; fecal beta-glucuronidase and beta-glucosidase activity (spectrophotometry); fecal pH and moisture. |
|  | Outcome Classification | Fecal microbiota composition (bacterial counts), fecal enzyme activity, SCFAs, and pH = prespecified primary outcomes per ClinicalTrials.gov (NCT00893659) |
|  | Statistical Analysis | Repeated-measures ANOVA (parametric) and Friedman test (non-parametric); paired t-test or Wilcoxon for within-group comparisons; α=0.05; analyses conducted in SPSS. |
|  | FDR / Multiple Testing Correction | No global multiplicity correction described; Bonferroni adjustment noted for some repeated-measures analyses (table footnotes). |
| (So et al., 2021) | Sample Collection | 3–4 g fecal material placed into tubes containing stabilization buffer; held at 4°C before collection; transported on ice within 24 h; stored at −80°C until analysis. |
|  | Biospecimen Processing / Extraction | Microbial DNA extracted using QIAamp DNA Stool Mini Kit (Qiagen); DNA stored at −20°C before sequencing . |
|  | Sequencing / Profiling Platform | 16S rRNA gene sequencing; V4 region (515F–806R primers); PCR in duplicate (30 cycles); Ion Torrent PGM platform . |
|  | Bioinformatic Processing | Metagenomic data analyzed using mothur; METASTATS used to assess compositional differences; absolute abundance values analyzed; diversity metrics computed in mothur . |
|  | Microbiome / Molecular Outcomes Assessed | Alpha diversity (observed species, Shannon index); beta diversity; Firmicutes/Bacteroidetes ratio; absolute abundance of selected taxa . |
|  | Outcome Classification | Changes in bacterial diversity, abundance of selected taxa, and F/B ratio = prespecified primary outcomes . |
|  | Statistical Analysis | Linear regression and multiple regression (sex-adjusted) to assess inter-group differences; two-sided tests; α=0.05 |
|  | FDR / Multiple Testing Correction | No multiple testing correction described |
| (Zhang et al., 2023) | Sample Collection | Fresh-frozen stool collection; OMNIgene GUT kit used when in-clinic collection not feasible; stool and plasma aliquots stored at −80°C until analysis. |
|  | Biospecimen Processing / Extraction | Bacteria DNA extracted using MagAttract PowerSoil kit (Qiagen). |
|  | Sequencing / Profiling Platform | Microbiome: 16S rRNA gene sequencing; V4 region; Illumina MiSeq and NovaSeq 6000; shotgun metagenomics in subset . Metabolomics: untargeted UPLC–qTOF (Waters Acquity UPLC, 2D column regeneration I-class/H-class, Xevo G2-XS) |
|  | Bioinformatic Processing | 16S: demultiplexing and quality filtering (USEARCH); OTU clustering at 97% similarity (UPARSE); taxonomy assigned using SILVA v128 (V4-trimmed); diversity calculated using ATIMA2. Shotgun: quality trimming (BBMap); taxonomic profiling (MetaPhlAn3); functional pathway profiling (HUMAnN3); UniRef90 regrouped to KEGG pathways. |
|  | Microbiome / Molecular Outcomes Assessed | Alpha diversity (Shannon, inverse Simpson); beta diversity (weighted Jaccard); relative abundance of taxa; metagenomic pathway abundance; circulating metabolites |
|  | Outcome Classification | Gut microbiome and circulating marker/metabolite profiles prescpecified as primary outcome; ClinicalTrials.gov ID: NCT02843425 . |
|  | Statistical Analysis | Generalized linear mixed models (random intercept); log transformation where appropriate; PERMANOVA for beta diversity; MaAsLin2 for taxa-level analyses; correlation analyses using diffcoexp framework. |
|  | FDR / Multiple Testing Correction | Benjamini–Hochberg FDR used when multiplicity relevant; q<0.20 threshold . |
| (Djuric et al., 2018) | Sample Collection | Unprepped colonic mucosal biopsies obtained via flexible sigmoidoscopy; flash-frozen in liquid nitrogen within ~5 seconds; stored at −70 °C. |
|  | Biospecimen Processing / Extraction | DNA isolated using PowerMag Microbiome RNA/DNA Isolation Kit with automated handling. |
|  | Sequencing / Profiling Platform | 16S rRNA gene sequencing; V4 region (515F/806R primers); dual-indexed libraries; Illumina MiSeq (Reagent Kit v2; 500 cycles). |
|  | Bioinformatic Processing | Mothur v1.35.0 following MiSeq SOP; quality filtering; chimera removal; OTU clustering at 97% similarity; taxonomy assignment using modified RDP training set; Yue & Clayton (YC) distance calculated for beta diversity. |
|  | Microbiome / Molecular Outcomes Assessed | Alpha diversity (Shannon, inverse Simpson); beta diversity (YC distance; PCoA); relative abundance of mucosal taxa. |
|  | Outcome Classification | ClinicalTrials.gov: NCT00475722 reports primary outcome as adherence to dietary goals. Mucosal microbiota composition and diversity were tertiary outcomes of parent trial not listed on ClinicalTrials.gov. |
|  | Statistical Analysis | LEfSe for differential taxa; Kruskal–Wallis and Jonckheere–Terpstra tests; Spearman correlations; regression analyses |
|  | FDR / Multiple Testing Correction | Benjamini–Hochberg adjustment applied; FDR-adjusted p-values reported for taxa and diversity analyses. |
| (Griffin et al., 2019) | Sample Collection | Fasting blood collected in EDTA tubes; serum stored at −80 °C. Unprepped colonic mucosal biopsies obtained by flexible sigmoidoscopy; flash-frozen; stored at −70 °C. |
|  | Biospecimen Processing / Extraction | Bacterial DNA isolated from colon biopsies; serum prepared for metabolite quantification. |
|  | Sequencing / Profiling Platform | 16S rRNA gene sequencing; V4 region; Illumina MiSeq; subsampled to 1,047 sequences per sample; genus-level classification using modified RDP training set in mothur. Serum metabolites (TMAO, choline, carnitine, betaine, γ-butyrobetaine) quantified by UPLC–MS/MS. |
|  | Bioinformatic Processing | Mothur pipeline; diversity indices calculated (Shannon, inverse Simpson); community distance (Yue & Clayton θYC); mean inter-individual θYC calculated per time point. |
|  | Microbiome / Molecular Outcomes Assessed | Alpha diversity (Shannon, inverse Simpson); beta diversity (θYC distance); relative abundance of mucosal taxa (including Akkermansia mucinophilia); circulating TMAO and related metabolites. |
|  | Outcome Classification | ClinicalTrials.gov: NCT00475722 reports primary outcome as adherence to dietary goals. Primary objective of parent RCT as reported in manuscript: colonic eicosanoid concentrations. Microbiome and circulating TMAO analyses were tertiary outcomes of parent trial not listed on ClinicalTrials.gov |
|  | Statistical Analysis | Mixed-models ANOVA for longitudinal changes (covariates: age, smoking); Spearman correlations for associations; chi-square and t-tests for group comparisons |
|  | FDR / Multiple Testing Correction | Benjamini–Hochberg false discovery rate applied where appropriate (e.g., correlation analyses). |
| (Umoh et al., 2016) | Sample Collection | Fasting serum and plasma collected; aliquots stored at −80 °C. |
|  | Biospecimen Processing / Extraction | Serum prepared for ELISA and GC–MS analyses; acid hydrolysis performed to release bacterial fatty acids prior to derivatization. |
|  | Sequencing / Profiling Platform | Not applicable (no microbiome sequencing performed). |
|  | Bioinformatic Processing | Not applicable. |
|  | Microbiome / Molecular Outcomes Assessed | Serum lipopolysaccharide-binding protein (LBP) by ELISA; branched-chain bacterial fatty acids (i15:0, i16:0, i17:0) quantified by GC–MS |
|  | Outcome Classification | ClinicalTrials.gov: NCT00475722 reports primary outcome as adherence to dietary goals. LBP and bacterial fatty acids were tertiary outcomes of parent trial not listed on ClinicalTrials.gov |
|  | Statistical Analysis | Linear mixed regression models for time and diet effects (age-adjusted); Spearman correlations; multiple linear regression with backward selection |
|  | FDR / Multiple Testing Correction | No global FDR adjustment; Bonferroni used for selected post hoc comparisons. |
| (Baxter et al., 2018) | Sample Collection | Self-collected stool; delivered to laboratory within 24 h; stored at −20°C; lyophilized, homogenized, aliquoted, and stored at −80°C prior to metabolomic extraction. |
|  | Biospecimen Processing / Extraction | Non-targeted metabolite extraction using 80% methanol; prepared for GC–MS and UPLC–MS/MS analyses. |
|  | Sequencing / Profiling Platform | Non-targeted metabolomics profiling by GC–MS and UPLC–MS/MS (positive and negative ion modes) |
|  | Bioinformatic Processing | Metabolite identification and relative abundance quantification; median scaling applied; pathway enrichment scores calculated; pathway visualization performed using Metabolync |
|  | Microbiome / Molecular Outcomes Assessed | Stool metabolite profiles |
|  | Outcome Classification | Stool metabolome modulation = prespecified primary outcome per ClinicalTrials.gov (NCT01929122). |
|  | Statistical Analysis | Matched-pairs 2-way ANOVA |
|  | FDR / Multiple Testing Correction | Benjamini–Hochberg FDR used; q<0.10 threshold . |
| (Zarei et al., 2021a) | Sample Collection | Fasting plasma (EDTA tubes) and first-morning urine collected; samples stored at −80°C until analysis. |
|  | Biospecimen Processing / Extraction | Non-targeted metabolite extraction using 80% methanol; prepared for UHPLC-MS/MS analyses. |
|  | Sequencing / Profiling Platform | Non-targeted metabolomics profiling of plasma and urine by UHPLC-MS/MS (positive and negative ion modes) |
|  | Bioinformatic Processing | Metabolite identification; median scaling applied; pathway assignment |
|  | Microbiome / Molecular Outcomes Assessed | Plasma and urine metabolite profiles |
|  | Outcome Classification | Plasma and urine metabolome modulation = prespecified primary outcome per ClinicalTrials.gov (NCT01929122). |
|  | Statistical Analysis | Welch two-sample t test and two-way ANOVA for between-group comparisons. Repeated-measures ANOVA for within-group time comparisons |
|  | FDR / Multiple Testing Correction | Benjamini–Hochberg FDR used; q<0.20 threshold. |
| (Brown et al., 2017) | Sample Collection | Self-collected stool; lyophilized; aliquoted; stored at −80°C prior to metabolomic extraction. |
|  | Biospecimen Processing / Extraction | Non-targeted metabolite extraction using 80% methanol. |
|  | Sequencing / Profiling Platform | Non-targeted metabolomics profiling by GC–MS and UPLC–MS/MS (positive and negative ion modes) |
|  | Bioinformatic Processing | Metabolite identification and relative abundance quantification; median scaling applied; pathway enrichment scores calculated using Metabolync |
|  | Microbiome / Molecular Outcomes Assessed | Stool metabolite profiles |
|  | Outcome Classification | Stool metabolome modulation = prespecified primary outcome per ClinicalTrials.gov (NCT01929122). |
|  | Statistical Analysis | Matched-pairs two-way ANOVA |
|  | FDR / Multiple Testing Correction | Benjamini–Hochberg FDR used; q<0.10 threshold |
| (Sheflin et al., 2017) | Sample Collection | Self-collected stool; transported within 24 h; stored at −20°C to −80°C |
|  | Biospecimen Processing / Extraction | DNA extracted using MoBio PowerSoil DNA Isolation Kit; stool metabolites prepared for SCFA and bile acid targeted analyses |
|  | Sequencing / Profiling Platform | 16S rRNA gene sequencing; V3–V5 region; SCFAs quantified by GC-FID; bile acids and phenolic acids measured by LC-MS. |
|  | Bioinformatic Processing | Sequence quality filtering and OTU clustering (99% similarity) using mothur; diversity metrics calculated; differential taxa identified using METASTATS; functional prediction using PICRUSt with KEGG pathway analysis in STAMP. |
|  | Microbiome / Molecular Outcomes Assessed | Alpha diversity (observed OTUs, Chao1, Simpson); relative abundance of taxa; predicted KEGG functional pathways; fecal SCFAs; bile acids; phenolic acids. |
|  | Outcome Classification | Primary outcomes not prespecified |
|  | Statistical Analysis | t-tests and Wilcoxon tests for within- and between-group comparisons; PCA for community structure analysis |
|  | FDR / Multiple Testing Correction | No global FDR adjustment reported. |
| (Byrd et al., 2025) | Sample Collection | Rectal biopsies immediately frozen without fixative |
|  | Biospecimen Processing / Extraction | Enzymatic lysis and bead homogenization (Bead Ruptor); DNA isolated using AutoGen Animal Tissue DNA Extraction Kit. |
|  | Sequencing / Profiling Platform | 16S rRNA gene sequencing; V4 region; 30 PCR cycles; 2×250 bp paired-end; Illumina MiSeq v2 (500-cycle kit). |
|  | Bioinformatic Processing | DADA2 v1.2.1 for ASV inference (100% sequence variants); taxonomy via SILVA v123; non-bacterial reads removed; diversity metrics calculated in QIIME v1.9.1; rarefied to 8,000 reads. |
|  | Microbiome / Molecular Outcomes Assessed | Alpha diversity (Observed ASVs, Shannon, Faith’s PD); beta diversity (Bray–Curtis, weighted/unweighted UniFrac); relative abundance of pre-specified and exploratory taxa. |
|  | Outcome Classification | Microbiome outcomes = secondary/ancillary analysis; parent RCT primary endpoint = colorectal adenoma recurrence |
|  | Statistical Analysis | Repeated-measures linear mixed-effects models with participant random intercept; intervention×time interaction tested via likelihood ratio tests; PERMANOVA (vegan adonis, stratified by participant); two-sided α=0.05. |
|  | FDR / Multiple Testing Correction | No correction in primary analyses; Bonferroni applied for exploratory taxa-level testing |
